# Supplementary material for: α-Acylamino-β-lactone N-Acylethanolamine-hydrolyzing Acid Amidase Inhibitors Encapsulated in PLGA Nanoparticles: Improvement of the Physical Stability and Protection of Human Cells from Hydrogen Peroxide-Induced Oxidative Stress
Source: Antioxidants (Basel). 2022 Mar 31;11(4):686. doi: 10.3390/antiox11040686 (PMC9028182; doi:10.3390/antiox11040686)
Supplement: Supplementary file 1 [file antioxidants-11-00686-s001.zip › antioxidants-1620715-supplementary.pdf]

α-Acylamino-β-lactone *N*-acylethanolamine-hydrolyzing acid amidase inhibitors encapsulated in PLGA nanoparticles: improvement of the physical stability and protection of human cells from hydrogen peroxide-induced oxidative stress

Agnese Gagliardi[a], Roberto Molinaro[b], Massimo Fresta[a], Andrea Duranti[c*], Donato Cosco[a*]

[a]Department of Health Sciences, University "Magna Græcia" of Catanzaro, Campus Universitario "S.Venuta", I-88100 Catanzaro, Italy

[b]IRCCS Ospedale San Raffaele srl, I-20132 Milan, Italy

[c]Department of Biomolecular Sciences, University of Urbino Carlo Bo, Piazza del Rinascimento, 6 61029 Urbino (PU), Italy

Supplementary Material

Table S1. Physico-chemical properties of PLGA nanoparticles (2.4 mg/mL) containing URB866.

URB866 (mg/mL)	Size (nm)	Polydispersity Index	Zeta Potential
-	120 ± 1	0.08 ± 0.01	-23 ± 1
0.1	147 ± 1	0.06 ± 0.03	-26 ± 1
0.2	175 ± 1	0.09 ± 0.01	-28 ± 1
0.4	180 ± 2	0.16 ± 0.02	-24 ± 1

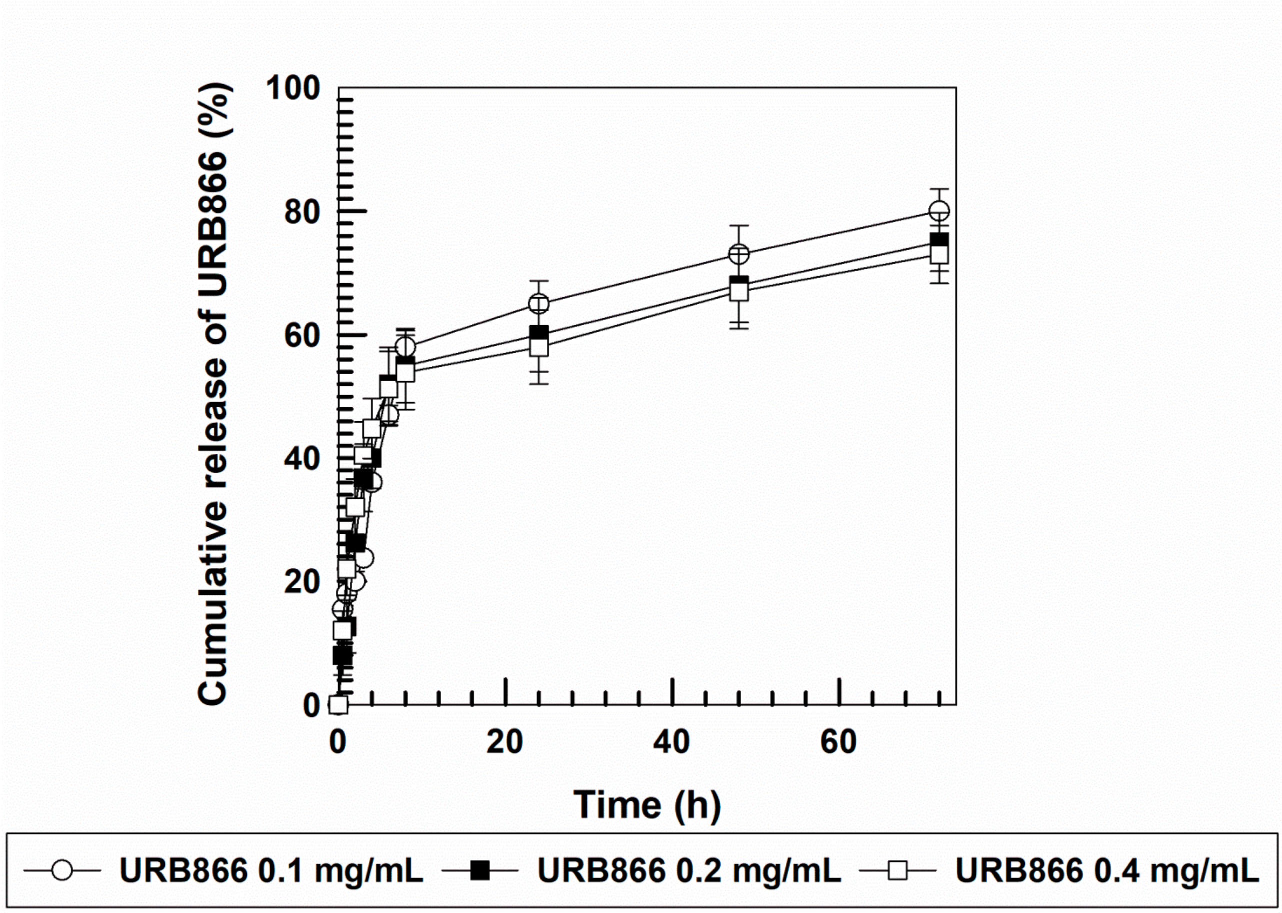

Figure S1. Release profile of URB866 from PLGA nanoparticles as a function of the entrapped drug and incubation time. Values represent the mean of three different experiments ± standard deviation.
